# Quantitative analysis of global protein stability rates in tissues

**DOI:** 10.1038/s41598-020-72410-y

**Published:** 2020-09-29

**Authors:** Daniel B. McClatchy, Salvador Martínez-Bartolomé, Yu Gao, Mathieu Lavallée-Adam, John R. Yates

**Affiliations:** 1 Department of Molecular Medicine, The Scripps Research Institute, La Jolla, CA USA; 2 College of Pharmacy, University of Illinois at Chicago, Chicago, IL USA; 3 Department of Biochemistry, Microbiology and Immunology and Ottawa Institute of Systems Biology, University of Ottawa, Ottawa, ON Canada

**Keywords:** Biological techniques, Biotechnology, Neuroscience, Diseases

## Abstract

Protein degradation is an essential mechanism for maintaining proteostasis in response to internal and external perturbations. Disruption of this process is implicated in many human diseases. We present a new technique, QUAD (Quantification of Azidohomoalanine Degradation), to analyze the global degradation rates in tissues using a non-canonical amino acid and mass spectrometry. QUAD analysis reveals that protein stability varied within tissues, but discernible trends in the data suggest that cellular environment is a major factor dictating stability. Within a tissue, different organelles and protein functions were enriched with different stability patterns. QUAD analysis demonstrated that protein stability is enhanced with age in the brain but not in the liver. Overall, QUAD allows the first global quantitation of protein stability rates in tissues, which will allow new insights and hypotheses in basic and translational research.

## Introduction

Proteostasis is the coordinated regulation of many cellular processes, including protein synthesis, degradation, and folding, to maintain a fully functional proteome in response to cellular perturbations. Dysfunction in any of these cellular processes can disrupt the proteome and trigger disease. Protein degradation is required to maintain optimal protein concentrations in response to changes in the cellular environment, and to prevent the accumulation of damaged proteins^1^. Autophagy and the ubiquitin proteasome system are two main processes that regulate protein stability in a cell. Both are tightly regulated, and dysfunction has been linked to various human diseases^2,3^. Global protein stability rates, are generally measured a “pulse-chase” experiment, where proteins are labeled or tagged and then quantitated by the loss of protein signal with time using immunoblots, fluorescence, or mass spectrometry(MS)^4–11^. However, these techniques are primarily used on cultured cells because they are difficult to employ in tissue. As a result, there are very few reports of quantitation of protein stability rates in tissues. In one report, rats were fully labeled with heavy nitrogen (^15^N) through an ^15^N diet, and protein stability was measured after switching rats to a normal ^14^N diet^12^. After 6 months on the ^14^N diet some proteins that were still labeled with ^15^N were detected by MS, suggesting that these proteins are either very stable, or extreme long-lived proteins (ELLP). Protein stability studies of tissue using the ^15^N labeling ELLP strategy^13^ identified proteins that persisted many months. A few other tissue stability studies have reported global protein turnover rates^14–17^. To calculate these fractional protein turnover rates, a pool of natural unlabeled proteins is chased out by isotopically labeled proteins or vice versa. By measuring both synthesis and degradation of peptides, mathematical modeling is used to calculate degradation rates or protein half-lives under steady-state conditions. When the system is perturbed (i.e. pathological conditions), it can be difficult to determine if synthesis or stability are responsible for changes in protein turnover rates using this strategy^16^.

We propose to use azidohomoalanine (AHA) to directly quantitate protein stability rates in tissues using pulse-chase labeling coupled with MS, which will provide better temporal resolution than the ELLP strategy. AHA is a non-canonical amino acid(ncAA) that can be inserted into proteins in vivo because it is accepted by the endogenous methionine tRNA synthetase. Using click chemistry, AHA containing proteins can be covalently bound to a biotin-alkyne and the AHA containing proteins can then be enriched with neutravidin beads. The use of AHA was originally described in the BONCAT(Biorthogonal Non-canonical Amino acid Tagging) method which labeled cultured cells for short time periods to identify newly synthesized proteins using MS^18^. One concern about employing ncAAs is whether they perturb protein function and/or structure. However, over the decade of biological studies employing AHA, no toxicity has been reported, suggesting little to no perturbation to native protein characteristics. This mostly likely stems from the fact that AHA structure is so remarkably similar to methionine that it interacts with the endogenous tRNA synthetase. In fact, AHA has been employed as an infrared spectroscopy probe to study native protein structure and folding^19–21^. Numerous biological studies have used AHA to study the most delicate and fragile proteomes with no disruption of function^22–24^. Most relevant to this study, AHA labeled proteins have been shown to be as stable as native proteins in cultured cells^25,26^. However, many of these studies briefly deplete or restrict the proteome of Met, which is an essential AA. Thus, validation of discoveries using AHA technology are needed in a native proteome as some studies have reported^27,28^. Using PALM (Pulse AHA Labeling in Mammals), it has recently been demonstrated that AHA can be safely incorporated into the proteomes of mouse tissues through their diet to identify newly synthesized proteins^29^. In this study, we Quantification of AHA Degradation (QUAD) in labeled proteins to measure protein stability rates in tissues.

## Results

### AHA pulse-chase strategy to quantitate global protein stability rates in tissues

**Figure 1:**
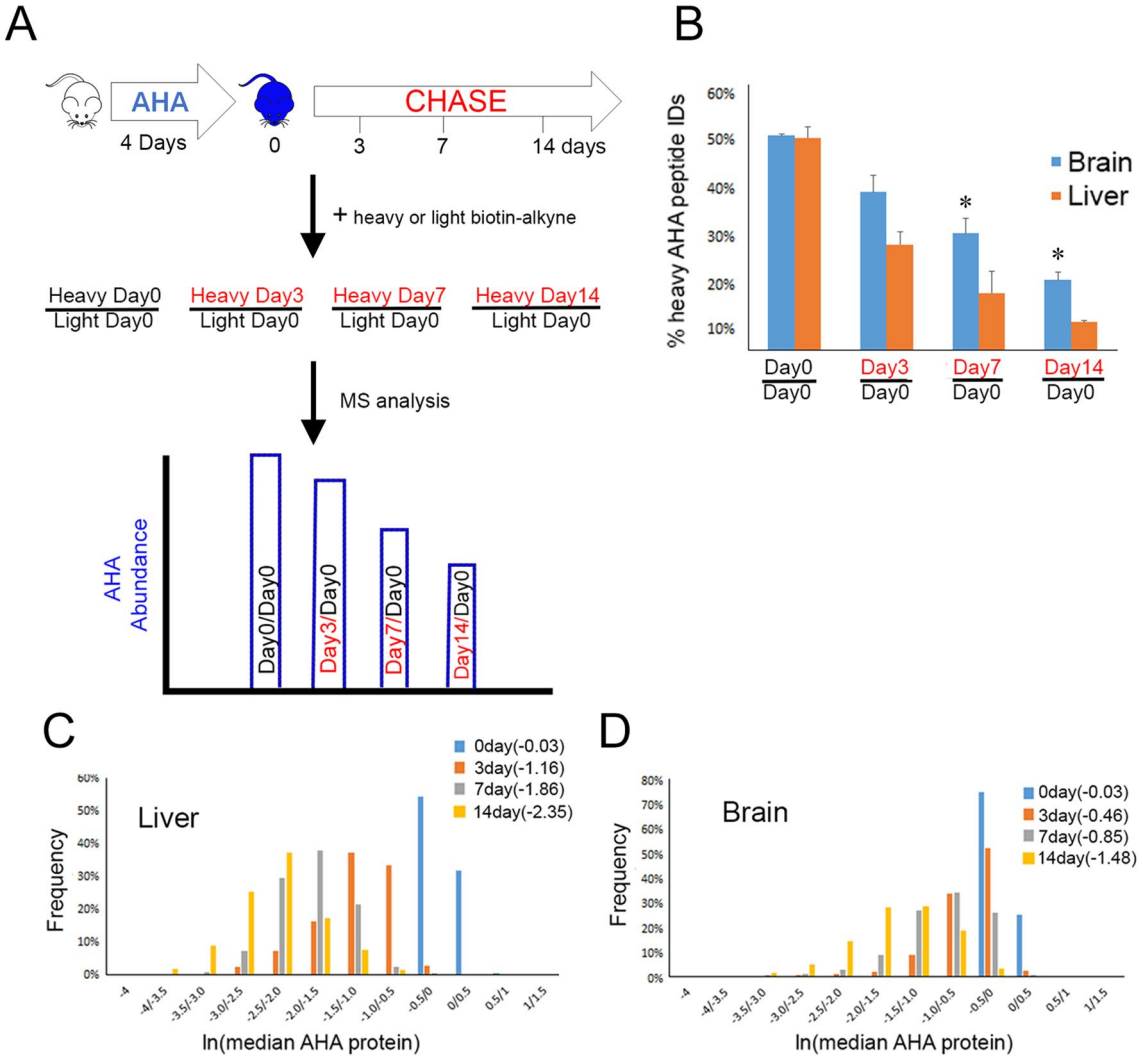
(**A**) Schematic of the experimental MS design. (**B**) The number of AHA peptides identified from chase time points decreases with increased chase time. Percentages of heavy AHA peptide identified (y-axis) from the total AHA identifications (i.e. light plus heavy) were calculated from MS analysis of different sample mixtures. The Day0/Day0 consists of two MS analyses of technical replicates and other mixtures represent three MS analyses from three mice. Liver tissue had significantly (* p < 0.05) fewer heavy AHA peptide identifications than brain tissue at Day7 and Day14 using a two-tailed t-test at each chase point. The abundance of the AHA proteins decreases with chase time in both liver (**C**) and brain(**D**). The median AHA peptide heavy/light ratios were calculated for each protein at each chase time point. After a natural log transformation, they were plotted in a histogram with the y-axis representing the percentage of proteins. The median protein ratio for each time point is in parentheses in the legend.

Figure 1A illustrates the QUAD workflow. Twelve one-month old male C57BL/6 mice were placed on an AHA diet as previously described^29^. After 4 days, three mice were sacrificed, and tissues were harvested. This group was designated Day0. The remaining mice were returned to a normal mouse diet for various “chase” times. Three mice were sacrificed after three (Day3), seven (Day7), and fourteen (Day14) days on a normal mouse diet. These timepoints were chosen because a previous study reported that the lifetime of a majority of proteins in the brain ranged between 3 and 13 days^30^. After tissue homogenization, click chemistry was performed to covalently add a biotin-alkyne to all AHA containing proteins. Immunoblot analysis demonstrated that biotin was observed at all time points, with the most observed at Day0 and the least at Day14 (Fig. S1A). For MS analysis, samples were labeled with either a light or heavy biotin-alkyne to enable quantification based on the calculation of heavy/light ratios^29^. Day0 samples were labeled with the light biotin-alkyne and all other time points were labeled with the heavy biotin-alkyne. The Day0 samples from different mice were combined to generate one internal standard and was mixed 1:1(wt/wt) with samples from individual mice at the other time points. As a baseline measurement, an aliquot of Day0 labeled with light biotin-alkyne was mixed 1:1 (wt/wt) with an aliquot of Day0 labeled with heavy biotin-alkyne. Next, the mixtures were digested with trypsin and the peptides that contained AHA were enriched with neutravidin beads. The enriched AHA peptides were eluted from the beads and analyzed by MS. MS analysis was first performed on liver and brain tissue samples. Peptide identification was limited to AHA-containing peptides. After data analysis, quantified AHA proteins were reported in four heavy/light mixtures: Day0/Day0, Day3/Day0, Day7/Day0, and Day14/Day0. Across all samples, over 500,000 non-unique AHA peptides were identified representing 6,614 protein groups.

The percentage of heavy AHA peptides identified in each MS analysis was calculated (Fig. 1B). For the baseline (i.e. Day0-Heavy/Day0-Light), ~ 50% of the AHA peptides identified were heavy. With a longer chase time, the percentage of heavy AHA peptides identified decreased. Although the baseline was similar between liver and brain, the percentage of heavy AHA peptides identified in the liver was significantly less than in the brain at Day7 and Day14. Since the ability to identify a peptide in the mass spectrometer is directly related to its abundance in the sample, this suggests that heavy AHA proteins become less abundant with longer chase times. The heavy AHA proteins identified at each time point in all three biological replicates were assigned to a large variety of functions, indicating the QUAD strategy is capable of a global analysis of protein stability (Fig. S1B).

**Figure 2:**
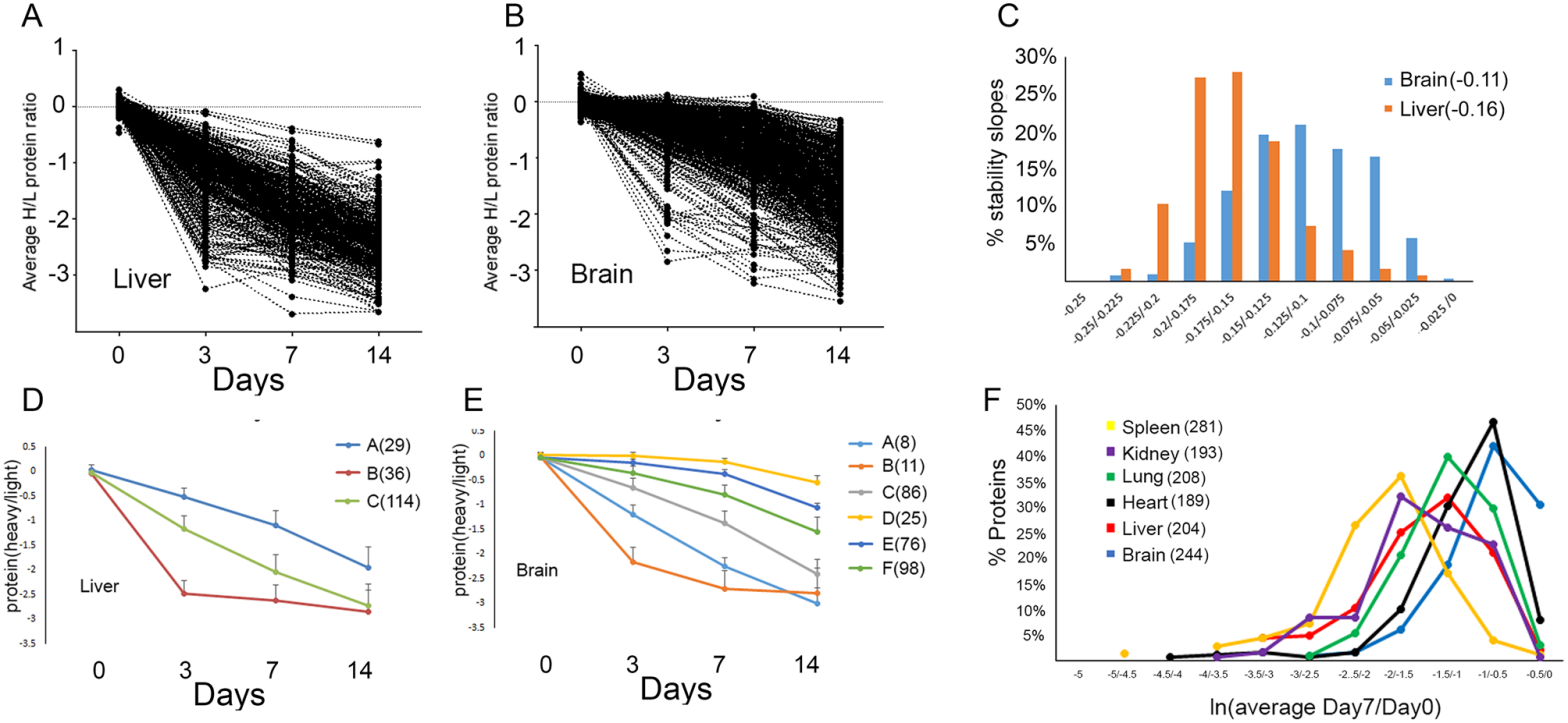
PST are calculated for liver(**A**) and brain(**B**) using average AHA protein ratio for three mice at each chase time point. All data has been natural log transformed. (**C**) The distribution of the slopes of the PST is unique for each tissue. The slopes were calculated for the PST reported in Figs. 2A and 2B and plotted in a histogram with the percentage of slopes on the y-axis. The average slope for each tissue is in parentheses and was significantly (p < 0.0001) different using a two-tail t-test. Clustering analysis was performed on the PST from liver (**D**) and brain (**E**) separately to identify global trends in each tissue. For each cluster, the average protein heavy/light ratio and standard deviation at each time point was plotted. The number of proteins in each cluster is in parentheses in the legend. **F**, Quantitative differences between the average Day7/Day0 AHA protein averages of multiple tissues from three mice. One-way ANOVA analysis resulted a p-value of 0.0003. Asterisks indicate the p-values from Bonferroni’s post-hoc test between individual tissues. Brain was significantly different from liver**, spleen*** and kidney** and heart was also significantly different from liver*, spleen**, and kidney*. *p < 0.05, **p < 0.01, ***p < 0.001.

The abundance of the heavy and light AHA peptides were quantified to generate heavy/light ratios. On average, 8.3 peptides were quantified per protein. The correlation between biological replicates suggested good reproducibility, and thus, accurate quantification (Fig. S2). As the chase time became longer, the heavy/light protein ratios became progressively smaller for both tissues (Fig. 1C,D). However, the median ratio in the liver was consistently smaller than the median ratio in the brain at all chase time points. Next, an average “protein stability trajectory” or PST was graphed for each AHA protein that was quantified at all chase time points. In total, 617 and 407 PSTs were determined for brain and liver, respectively. A majority of the proteins followed a linear continuous decay, but a smaller subset exhibited non-exponential decay, as previously described^25^. A wide distribution of trajectories was observed in both tissues, but more trajectories from the liver appeared to have a steeper slope than from the brain, (Fig. 2A,B; Table S1 and S2). To further investigate, the slope was calculated for each PST. A slope of zero would indicate no change in AHA protein abundance over time (i.e. very stable). The average brain slope (−0.11) was significantly (p < 0.0001) different from liver (−0.16) (Fig. 2C). In the brain, the myelin basic protein(MBP), sirtuin-2, and 2′,3′-cyclic-nucleotide 3′-phosphodiesterase(CNP) were among the most stable proteins while cofilin-1 was one of the least stable (Fig. S1C), which is consistent with previous reports^12,15^. We found no evidence in the literature that slopes were previously calculated to extract protein stability information; instead, published protein tissue turnover papers calculated protein half-lives. The protein half-lives of the liver and brain datasets in Fig. 2A,B were calculated and compared to the slope. There was a significant correlation (r = 0.84, p < 0.0001) between the two measurements (Fig. S1D, Table S3). Finally, we tested whether any intrinsic protein characteristics could contribute to the differences in PST. There was no correlation between slope and molecular weight (Fig. S3A), abundance (Fig. S3B), or transmembrane regions (Fig. S3C). Examination of three different databases of intrinsic protein disorder revealed a significantly negative correlation between protein disorder and slope, which is consistent with the hypothesis that increases in protein disorder correspond to decreased protein stability or decreased slope (Fig. S3D, Fig. S3E, and Fig. S3F)^31^.

### Tissues differentially regulate protein stability

PSTs were further analyzed by an unsupervised learning approach (i.e. clustering analysis), where representative clusters were determined based on the average slope and shape trajectory. To increase confidence in our dataset, to qualify for further analysis the PSTs of each biological replicate for each protein were required to cluster together. This removed the few spurious PSTs that did not exhibit a linear or non-exponential decay. There were six distinct clusters for brain, and three for liver (Fig. 2D,E; Table S4 and Table S5). Although some clusters had the same endpoint, the route to that endpoint was different, as illustrated by liver cluster B and C. Clusters with shallow slopes (i.e. cluster D, E, and F) were unique to brain tissue. For a direct comparison, clustering analysis of PSTs was performed on one dataset containing both the liver and brain. Four clusters were clearly distinguishable (Fig. S4A; Table S6). Proteins from both tissues were present in all clusters, but the clusters were biased towards one tissue (Fig. S4B). Most liver proteins were assigned to clusters with steep slopes whereas most brain proteins were assigned to the clusters with shallow slopes. Almost all identical proteins that were quantified in both liver and brain tissue were less stable in the liver compared to the brain (Fig. S4C; Table S7), but no proteins were observed to be less stable in the brain than in the liver. Only serum and blood proteins exhibited no stability differences between liver and brain (Fig. S4D). Thus, comparison of proteins stability in liver and brain suggests that it can be a defining trait of tissues. To further explore how tissues can influence protein stability, a second QUAD dataset was generated with an additional three mice to analyze other tissues (kidney, heart, spleen, and lung) and a second analysis of liver and brain tissues. For this QUAD analysis, one chase time of 7 days was chosen for analysis because it had larger changes in protein stability than the 3 day chase, but more AHA proteins identified than the 14 day chase. Consistent with the previous QUAD dataset, brain tissue had a significantly higher average Day7/Day0 quantitated ratio than liver, indicating again that AHA proteins are less stable in the liver than in the brain (Fig. 2F; Fig. S5A; Table S8). The brain average ratio was also significantly higher than kidney and spleen tissues. Heart tissue had a similar stability profile as brain tissue with significantly higher average ratio than liver, spleen and kidney. Overall, QUAD analysis revealed distinct protein stability trends can define a tissue proteome.

### Subcellular localization and protein function can influence protein stability

**Figure 3:**
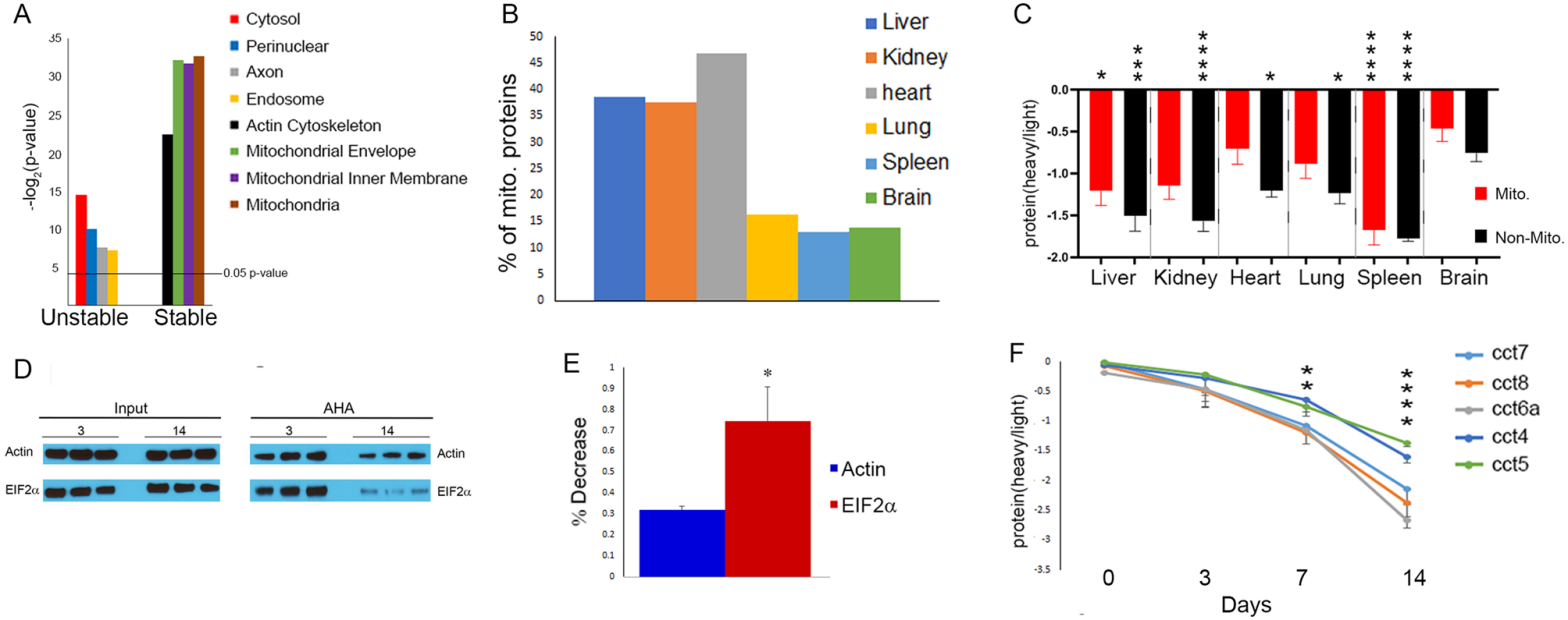
(**A**) GO analysis of stable and unstable protein datasets demonstrated significant enrichment of different subcellular components. The four most significantly enriched localizations are reported. Y-axis represents the negative log_2_ of p-value of enrichment. **B**) The percentage of mitochondrial proteins quantified in each tissue dataset is shown in Fig. 2F. (**C**) Re-analysis of the data in Fig. 2F. Comparison of the average Day7/Day0 protein ratio of mitochondrial (“mito” in red) and non-mitochondrial (“non-mito” in black) proteins in different tissues. One-way ANOVA analysis test computed a p-value of < 0.0001. All p-values from a post-hoc Tukey’s multiple comparisons test are in Table S9. The significant differences to the brain non-mitochondrial dataset from the Tukey’s test are shown in the figure. (**D**) Immunoblot analysis confirmed differences in stability between a translational (EIF2α) and a cytoskeletal (β-actin) protein. Click reaction was performed on brain homogenates from three mice at 3 day and 14 day. Samples were analyzed before (Input) and after (AHA) neutravidin enrichment. The images represent four separately processed immunoblots. The uncropped immunoblot images are in Fig. S8. (**E**) Significant difference between the stability of actin and EIF2α was observed with immunoblot analysis. Quantification of the pixel intensity of the immunoreactivity of the enrichment samples in Fig. 3D demonstrated a larger significant (p < 0.05*) difference between 3 and 14 day with EIF2α than with actin. The y-axis shows the percent decrease in immunoreactivity between 3 and 14 day. A two-tailed t-test was performed. (**F**) Significant differences in the stability of different TRIC complex subunits were observed in brain tissue. The average protein heavy/light ratio from biological replicates at each time point was plotted for subunits of the TRiC complex from brain tissue. One-way ANOVA analysis was performed on the subunit ratios at each time point. There was a significant difference observed at 7 day(p < 0.01 ) and 14 day(p < 0.0001). Multiple Comparison post-hoc test was performed to determine which subunits were significantly different. At 7 day, CCT4 vs CCT6a* and CCT4 vs CCT8*. At 14 day, CCT4 vs CCT6a***,CCT4 vs CCT7*, and CCT4 vs CCT8**, CCT5 vs CCT6a****, CCT5 vs CCT7**, and CCT5 vs CCT8***. Figures depict the Bonferroni's p-values. *p < 0.05, **p < 0.01, ***p < 0.001,****p < 0.0001.

We investigated whether protein stability is associated with any cellular characteristics within a tissue. For this analysis, the brain dataset pictured in Fig. 2E was employed because it contained a wide range of PST. Clusters D, E, and F were classified as “stable” proteins and clusters A, B, and C were classified as “unstable” proteins. GO enrichment analysis of subcellular compartments revealed mitochondrial compartments and actin cytoskeleton were enriched in the stable dataset, while cytosol, endosomes, axons, and perinuclear compartment were enriched in the unstable dataset (Fig. 3A). Since the number of mitochondria can vary between tissues, we tested whether the number of mitochondrial proteins found in different tissue datasets (Fig. 2F) accounted for the differences in protein stabilities observed in different tissues. Indeed, there were large differences in the proportion of mitochondrial proteins, ranging from 46.8% in the heart to 13.1% in the spleen (Fig. 3B). The tissue analysis was repeated, but this time we analyzed the mitochondrial and non-mitochondrial proteins separately (Fig. 3C and Table S9). The high percentage of mitochondrial proteins in the heart appeared partially responsible the greater stability observed in previous analysis. The brain proteome, however, appears to be more stable than other tissues regardless of how the data was analyzed.

It was determined whether any signaling pathway or cellular function was associated with protein stability. The most significantly enriched pathway in the unstable dataset (p value = 2.05 e^-16^) was protein metabolism, which included protein synthesis (Fig. S5A; Table S10). In contrast, the stable dataset was significantly enriched (p value = 1.43 e^-16^) in the cytoskeleton, which included cytoskeleton proteins and proteins that regulate the cytoskeleton (Fig. S5B; and Table S11). To provide further evidence for the results of the enrichment analysis, the degradation of a cytoskeleton protein (i.e. β-actin) was compared to the degradation of a translational protein (i.e. elongation initiation factor 2 alpha (EIF2α)) using antibodies (Fig. 3D). At 14 day immunoreactivity for both antibodies was decreased from 3 day, suggesting AHA protein degradation for both antibodies, but the decrease was significantly larger for EIF2α. β-actin decreased ~ 30% from 3 to 14 day while EIF2α decreased ~ 75% (Fig. 3E). However, there were significantly enriched pathway shared between the stable and unstable datasets. Proteins in these shared pathways showed many functional relationships between the stable and unstable proteins (Fig. S6), demonstrating that stable and unstable proteins can interact functionally in signaling networks.

### Protein complexes subunits share similar protein stability

To investigate whether unstable and stable proteins physically interact in vivo, the stable and unstable datasets were analyzed together using the database of mammalian complexes, CORUM^32^. Seventy-seven percent of the identified protein complexes contained either only stable proteins or only unstable proteins (Fig. S7A and Table S12). This is consistent with previous publications that reported that subunits of protein complexes possess similar protein turnover rates in mammalian tissues^15,30^. Protein complexes whose subunits possess a wide range of turnover rates have been identified (such as the Cop9 signalosome), but this is hypothesized to be related to distinct complex sub-populations with unique subunit compositions^15^. Interestingly, QUAD analysis revealed that the TRIC (TCP-1 Ring Complex) or complex chaperonin containing TCP1 complex (CCT) possess subunits with both stable and unstable subunits. The well-studied TRiC complex is a molecular chaperonin that consists of two ring structures, with each ring comprising eight subunits (CCT 1–8). The subunits are structurally similar, with an ATP-binding equatorial domain and an apical substrate-binding domain linked by an intermediate domain^33^. Statistical analysis confirmed that CCT6a, CCT7, and CCT8 are significantly less stable than the CCT4 and CCT5 subunits (Fig. 3F).

**Figure 4:**
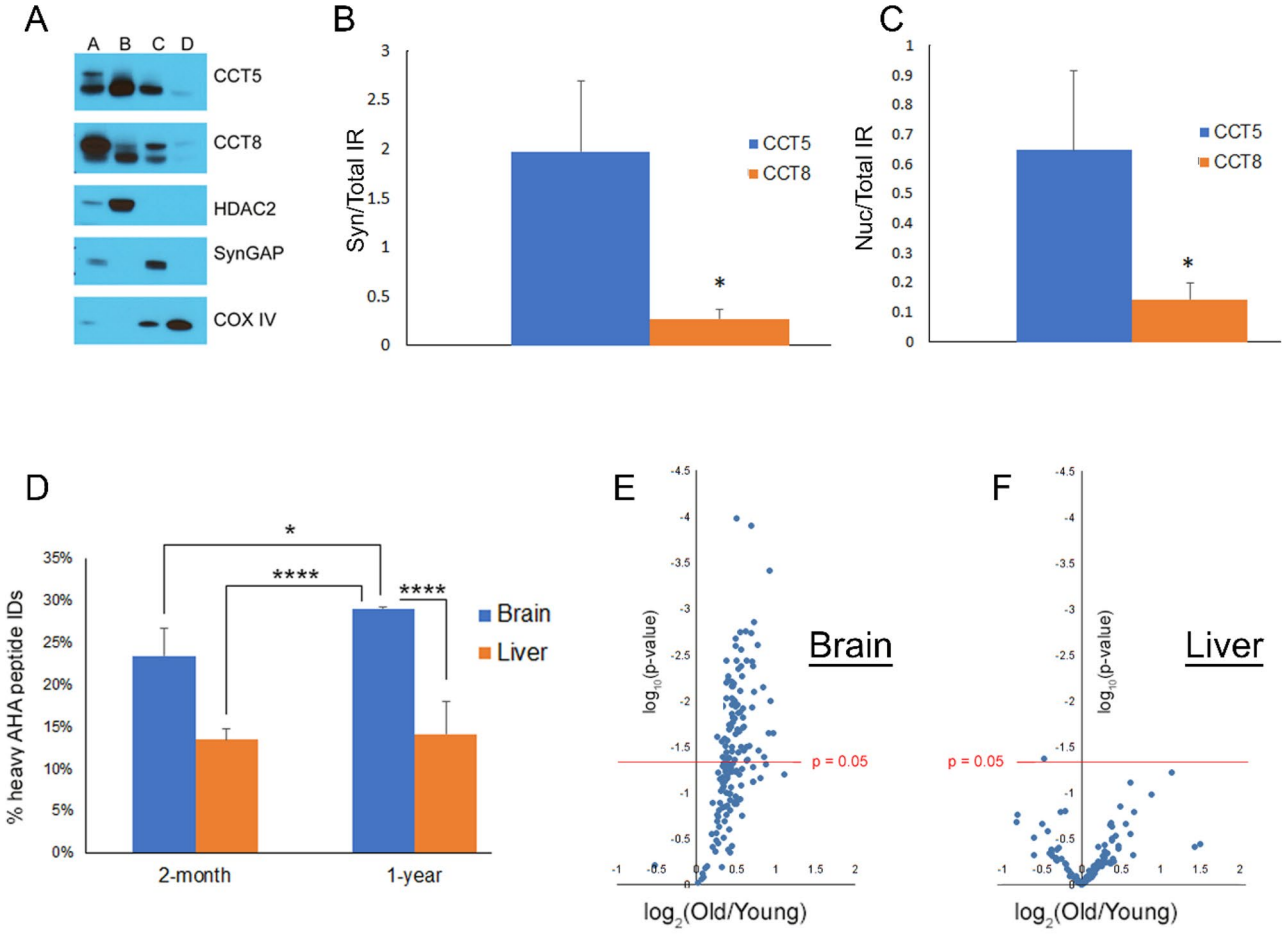
(**A**) Representative immunoblot of fractionated brain tissue. A = unfractionated brain, B = nuclear fraction, C = synaptosomal fraction, and D = mitochondrial fraction. Fractionation was validated by the enrichment of proteins known to reside in each compartment: HDAC2(nuclear protein), SynGAP(synaptic protein), and COX IV (mitochondrial protein). The images represent five separately processed immunoblots. The uncropped immunoblot images are in Fig. S9. (**B**) Quantitation of the CCT5 and 8 IR in the synaptosomal (**B**) and nuclear (**C**) fraction normalized to the CCT IR in unfractionated brain; N = 4. (**D)** The number of AHA peptides identified from a 7 day chase period was decreased in 2-month old brains compared with 1-year old brain. The average percentages of heavy AHA peptide identified (y-axis) from the total AHA identifications (i.e. light plus heavy) were calculated from MS analysis. N = 3. One-ANOVA analysis with Bonferroni’s post-hoc test was performed. Figure depicts the Bonferroni p-values for 1-year old brain. *p < 0.05, **p < 0.01, ***p < 0.001,****p < 0.0001. Bonferroni p-values not in the figure: 2mo-Brain vs. 2mo-Liver **, 2mo-Brain vs. 1 yr-Liver **, and 2mo-Liver vs. 1 yr-Liver not significant. (**E**) AHA proteins have a slower degradation rate in brains from 1-year old mice than 2-month old mice. (**F**) AHA proteins have similar degradation rate in livers from 1-year old mice than 2-month old mice. x-axis is the log_2_ fold change plotted as 1-year/2-month. Each point represents the average Day7/Day0 protein ratio, calculated from three mice in each age group. The y-axis is the log_10_ p-value with the red line representing the significant value filter 0.05.

Since the TRIC complex structure has not been studied in brain, we postulated that a non-canonical TRIC complex may exist in the brain. To provide additional evidence for non-canonical TRIC complexes, experiments were performed to determine if localization differences exist between stable and unstable CCT subunits. Commercial CCT antibodies failed to produce specific staining for immunohistochemistry(data not shown), so sucrose fractionation was employed to examine the nuclear, synaptosomal, and mitochondrial fractions^34^. CCT subunits have been identified in published MS proteomic datasets of these fractions^35–37^. We used immunoblots to compare the immunoreactivity (IR) of CCT5 and CCT8 in total brain homogenate to these fractions, and found that CCT5 was significantly more enriched than CCT8 in the synaptosomal and nuclear compartments (Fig. 4A–C). The CCT subunits were detected in the mitochondrial fraction, but in much lower abundance than the other fractions. Detection of mitochondrial CCT IR led to saturation of the CCT IR in the total homogenate, preventing quantitation of the immunoblots.

### Age influences protein stability in brain tissue

Proteostasis dysfunction has been implicated in age-related neurological disorders^38^. To assess how age affects protein stability, QUAD analysis was applied to brain and liver tissue from 1-year old mice and compared to the two-month old mouse dataset (Fig. 2F) using the Day7 chase time point. There was a higher percentage of heavy Day7 peptides in brain than in liver regardless of age. In addition, there was a significantly higher percentage of heavy Day7 peptides identified in 1-year old brains than in 2-month old brains, but there was no significant difference between 2-month old and 1-year old livers (Fig. 4D). Next, the AHA proteins that were quantified in three mice at 2-months and at 1-year were compared. At 1-year, the Day7/Day0 protein ratios were equal to or greater than the ratios of the 2-month old brains (Fig. 4E; Table S13), but these ratios were evenly distributed in liver (Fig. 4F; Table S14). Annotation of protein functions of the proteins significantly altered in brain with age revealed a wide variety of functional classes, most of which were also observed in the liver analysis (Fig. S7B). Furthermore, there were four proteins in the brain that were significantly altered with age; these proteins were quantified in the liver analysis but were not found to be significantly altered (Fig. S7C). Thus, this QUAD analysis demonstrates that global protein stability is enhanced with age in brain tissue, but age has no detectable effect on protein stability in liver tissue.

## Discussion

The QUAD method directly quantitates the loss of AHA proteins from tissue proteomes to accurately quantitate the stability of individual proteins. In contrast, previous publications have reported global protein turnover rates in tissues by measuring both increase of unlabeled peptides with the loss of identical labeled peptides or vice versa. These synthesis and degradation measurements data are used to calculate the protein turnover. Another difference between QUAD analysis and previous protein turnover studies is that the protein degradation rates were calculated using the slopes of the PSTs while the protein turnover studies calculate half-lives. However, it was demonstrated that slopes and half-lives calculations are highly correlated with each other. In addition, tissues have different AA incorporation rates which can interfere with comparisons between tissues^13^. The QUAD method ignores the variability of AA incorporation and protein synthesis and solely measures protein degradation rates in tissues. The nature of AHA offers a clear advantage of QUAD over protein turnover studies, because results can be verified by immunoblots as shown here and possibly by imaging using FUNCAT^39^. One important factor in protein turnover studies is the uptake and recycling of heavy and light AAs after protein degradation. However, mathematical models have helped reduce this problem in turnover calculations^30^, and the unique nature of AHA may further minimize it. After the AHA diet without Met, the mice are placed on normal Met containing mouse diet. Met affinity for the Met tRNA synthetase is more than 300 fold greater than AHA, and even a small amount of Met has been reported to decrease AHA incorporation^40,41^. Since the first timepoint was recorded at 3 days chase, uptake and recycling of AAs will have minimal or no effect on calculated stability, but it is still a caveat that should be taken into consideration, especially if examining timepoints < 3 days.

Nevertheless, our analysis using QUAD and the protein turnover studies both demonstrated that differences exist between tissues providing verification of our novel method. For example, Price et al. reported that the average protein turnover in liver was faster than in brain, similar to the findings of this study^15^. The difference between liver and brain protein stabilities is mostly likely due to the higher metabolic rate of liver than brain. This metabolic difference stems from liver being composed of dividing cells while the brain is a mixture of dividing (i.e. glia) and non-dividing cells(i.e. neurons)^13,42^. Our analysis also demonstrated that heart and brain tissue exhibit high protein stability compared to other tissues. Further analysis indicated that the high number of mitochondria in the heart was partly responsible for this high protein stability, but mitochondria did not appear to contribute to the high stability observed in brain tissue. Fornasiero et al. also reported that removing mitochondrial proteins from their protein turnover study revealed that brain was more stable than heart tissue^30^. This is strong evidence that the cellular environment is one of the main factors that determine protein stability and that measurement of degradation alone can define the uniqueness of tissue proteomes. Although the specifics of the cellular environment that contribute to a tissue characteristic degradation pattern is not entirely known, the presence of non-dividing cells is most likely an important trait as they are present in the most stable tissues (i.e. brain and heart). Further investigation, however, is required to fully elucidate the mechanism underlying these tissue specific protein stability patterns.

Within a tissue, there is also a large range of protein stabilities, and stability was not correlated with intrinsic protein characteristics, such as abundance, size, or structure (i.e. transmembrane regions). Price et al. did not observe any significant correlation between abundance and protein turnover, and while Fornasiero et al. found a correlation, it was a weak association (r^2^ = 0.11). Fornasiero et al. and our study both show a significant negative correlation between stability and intrinsic protein disorder, and both studies had similar weak correlation values (i.e. Fornasiero et al. r^2^ = 0.009 and this study r^2^ = 0.046). This supports the hypothesis that increased intrinsic protein disorder contributes to protein instability, but with a weak association, and it suggests that it has a minor influence^31^. In addition, our analysis observed that specific protein localizations and functions could be distinguished by protein stability, which has also been reported in tissue protein turnover studies^15,30^. Compared with the rest of the proteome, mitochondrial proteins were observed to be more stable. It is unclear if this is related to the mitochondrial function or the organelle microenvironment. Translational machinery was enriched in unstable proteins, and cytoskeletal proteins were enriched in stable proteins. Since the cytoskeleton provides the basis for cell polarity and intracellular transport, stability would be needed for these essential structural functions. Translation is just as essential for cellular function as the cytoskeleton, but stability of translation machinery may be deleterious to the cell since the overproduction of translational initiation factors is observed in cancer^43^. In animal models, it has also been reported that overexpression of initiation factors can increase the susceptibility to tumors, and reduction can suppress tumor development^44–47^. This suggests that tight control of translation factors through degradation is a crucial mechanism to prevent tumorigenesis.

It has been suggested that metabolism of coordinated subunits within a protein complex would prevent the presence of incomplete or non-functional complexes^15^. It also has been suggested that finding common degradation trends in the proteome may lead to the identification of new protein complexes^30^. Surprisingly, we detected significant stability differences between protein subunits of the TRIC chaperonin complex and postulated that this might indicate a non-canonical chaperonin in the brain. The identification of subunit differences in different subcellular compartments provides further evidence for this hypothesis. It has been reported that all subunits are required for chaperonin function, as deletion or mutation of any subunits is sufficient to impair the function of the chaperonin in cultured cells^48^. Detailed structure analyses have confirmed the existence of the eight-subunit chaperonin^49–51^, but there has been indirect evidence to suggest an alternative structure. This evidence includes the large difference in the subunit mRNA levels in mouse testes^52^ and brain regions^53^. In cultured cells, exogenously expressed subunits have been localized to different subcellular compartments^54–56^ and revealed subunit-specific phenotypes^54,57–59^. In summary, we believe the data demonstrates that the quantitation of protein stability rates in tissues can lead to new insights and hypotheses in basic and translational research.

Protein degradation is a crucial component of proteostasis, which has been postulated to decline with age. In C. elegans, protein turnover was reported to be slower in the adult worm than in the developing worm^60^, but no global studies in mammalian tissues could be found in the literature. Age-related decline of proteostasis in the brain has received considerable attention because age is a major risk factor for neurodegenerative diseases. A common pathological feature of these disorders is the accumulation and aggregation of misfolded proteins^61^. It has been hypothesized that age-related impairment of protein folding machinery leads to the observations of pathological misfolded proteins^62^. Our data suggests there is an age-related decline in protein degradation. Although this is the first study to quantify global degradation rates in tissues, other laboratories have demonstrated that chemical or genetic modification of autophagy or proteasomal degradation can affect neurological health in old mice. For example, induction of autophagy by rapamycin in vivo lowers intracellular amyloid beta levels and improves cognition^63^, and long-term rapamycin treatment reduces plaque load in Alzheimer’s disease mouse models^64^. Thus, we postulate that a global decline in protein degradation in brain tissue contributes to the vulnerability of the elderly to neurological diseases associated with protein misfolding.

In summary, deleterious changes in protein degradation have been implicated in diseases in almost every human tissue. QUAD analysis allows the global quantification of protein stability rates in any mouse tissue, which then can be extended to any mouse model of disease. Identification of changes in protein stability rates can precede detectable changes in the whole proteome, and possibly portend a disease phenotype. Interventions early in a disease have the greatest potential to prevent permanent damage to cells and tissues and early perturbations in the proteome may indicate that something is starting to go wrong. Thus, the temporal resolution of QUAD can identify alterations in protein stability prior to development of disease phenotypes, thus identifying potential targets to ameliorate or prevent pathogenesis. With the development of non-canonical amino acids with cell-type specificity^65^, AHA can be replaced to allow QUAD analysis to quantitate cell-specific protein stability in animal models of disease.

## Materials and methods

### Animals

Mice were housed in plastic cages located inside a temperature- and humidity-controlled animal colony and were maintained on a reversed day/night cycle (lights on from 7:00 P.M. to 7:00 A.M.). Animal facilities were AAALAC (Association for Assessment and Accreditation of Laboratory Animal Care ) approved, and protocols were in accordance with the IACUC(Institutional Animal Care and Use Committee). Male C57BL/6 1 month old mice were used for all experiments except those represented in Fig. 4E,F where 2-month and 1-year old mice were used. For the QUAD analysis, mice were fed the AHA diet for 4 days, as previously described^29^. AHA was purchased from Click Chemistry Tools (Scottsdale, AZ) and given to Envigo (Madison, WI) to manufacture the AHA mouse pellets. After 4 days, the mice were either sacrificed or returned to normal mouse feed for various times, as described in the Results section. Animals were anesthetized with halothane and sacrificed by decapitation. The whole tissues were quickly removed, dissected, and snap-frozen in liquid nitrogen.

### Tissue preparation

Tissues were prepared as previously described^29^. Briefly, the tissues were dissected into small pieces and homogenized at 4 °C using the Precellys 24 homogenizer in PBS with protease and phosphatase inhibitors (Roche,Indianapolis, Indiana). For fractionation, brain tissue was homogenized in a teflon dounce grinder on ice in PBS with protease and phosphatase inhibitors (Roche, Indianapolis, Indiana). After homogenization, protein concentration was determined with a Pierce BCA protein assay (Life Technologies, Grand Island, NY).

### Click chemistry

For MS analysis, 10 mg of each biological replicate plus 10 mg for the internal standard (Day0) were used, except in the experiments described in Fig. 4E,F where 5 mg were used for each biological replicate plus 5 mg for the internal standard (Day0). For immunoblot analysis (Fig. 3D), 4 mg of starting material was used. Sodium dodecyl sulfate was added to the homogenized tissues at final concentration of 0.5%. The homogenate was then sonicated with a tip sonicator and was divided into 0.5 mg aliquots. A click reaction was performed on each aliquot. The click reaction protocol has been previously published^66^. In brief, for each click reaction, the following reagents were added in this order: (1) 30 μL of 1.7 mM TBTA, (2) 8 μL of 50 mM copper sulfate, (3) 8 μL of 5 mM light biotin-alkyne (C_16_H_24_N_4_O_3_S, Seterah, Eugene, OR) or heavy biotin-alkyne (C_13_H_24_N_3_O_3_S^13^C_3_^15^N, Seterah, Eugene, OR), and (4) 8 μL of 50 mM TCEP. For the reaction described in Fig. 3D, biotin-PEG4-alkyne from Click Chemistry Tools (Scottsdale, AZ) was used. PBS was then added to a final volume of 400 μL and the reaction was incubated for 1 h at room temperature. The click reactions for each sample were combined and precipitation was performed with 25% TCA.

### Digestion and biotin peptide enrichment

Precipitated pellets were resuspended with MS-compatible surfactant ProteaseMAX (Promega, Madison, WI) and urea, then reduced, alkylated, and digested with TrypZean trypsin (St. Louis, MO, Sigma-Aldrich) at 1:25 dilution with the protein sample as previously described^29^. The digested solution was centrifuged at 13 000 g for 10 min. The supernatant was removed, and the pellet was resuspended with PBS and centrifuged at 13 000 g for 10 min. Supernatants were combined and 300 μL of neutravidin agarose resin (Thermo Fisher Scientific, Rockland, IL) was added. The resin was incubated with the peptides for 2 h at room temperature while rotating; then the resin was washed five times with PBS. The peptides were eluted four times with 250 µl 80% acetonitrile, 0.2% formic acid, and 0.1% TFA. The elutions were dried with a speed-vac and stored at − 80 °C until MS analysis.

### Mass spectrometry analysis

Enriched dried peptides were resolubilized in Buffer A (5% ACN, 95% water, 0.1% formic acid) and were pressure-loaded onto a 250-μm i.d. capillary with a kasil frit. The capillary contained 2 cm of 10 μm Jupiter C18-A material (Phenomenex, Ventura, CA), followed by 2 cm 5 μm Partisphere strong cation exchanger (Whatman, Clifton, NJ). This loading column was washed with buffer A. After washing, a 100 μm i.d. capillary with a 5 μm pulled tip packed with 15 cm 4 μm Jupiter C18 material (Phenomenex, Ventura, CA) was attached to the loading column with a union, and the entire split-column(loading column-union-analytical column) was placed in-line with an Agilent 1,100 quaternary HPLC (Palo Alto, CA). The sample was analyzed using an eleven step MudPIT, which is a salt-step separation previously described^22^. As peptides eluted from the microcapillary column, they were electrosprayed directly into a Velos mass spectrometer (ThermoFisher, Palo Alto, CA) with the application of a distal 2.4 kV spray voltage. A cycle of one full-scan FT mass spectrum (300 − 1,600 m/z) at 60 000 resolution followed by 20 data-dependent IT MS/MS spectra at a 35% normalized collision energy was repeated for each step of the multidimensional separation. For the analysis described in Fig. 4E,F, a nano-Easy HPLC (ThermoFisher) with an Elite mass spectrometer (ThermoFisher) was used with the MS settings previously described^22^.

### Analysis of mass spectra

MS1 and MS2 (tandem mass spectra) were extracted from the XCalibur data system format (.RAW) into MS1 and MS2 formats using RawExtract^67^. The MS2 files were interpreted by Prolucid and results were filtered, sorted, and displayed using the DTA Select 2 program using a decoy database strategy filtering for only fully tryptic peptides with a 5 ppm mass accuracy^68,69^. Searches were performed against UniProt mouse database released on 03-25-2014. No enzyme specificity was considered for any search. The following modifications were searched for: (1) static modification of 57.02146 on cysteine for all analyses, and (2) differential modification of 351.1774 (heavy) or 347.1702 (light) on methionine for AHA bound to a biotin-alkyne. The protein false discovery rate was < 1%. pQuant used the MS1 and DTASelect-filter files for the quantification of the heavy/light ratios using a 0.1 quality filter as previously described^29,70^. Proteins were only reported if at least one unique peptide was quantified. Redundant or subset proteins were not reported.

### Sucrose fractionation of brain tissue

Brain tissue was fractionated as previously described^34^. Briefly, whole brains were homogenized in 4 mM HEPES(pH 7.4), 0.32 M sucrose (i.e. Buffer H) using a Teflon dounce grinder. Homogenates were centrifuged at 800 × g at 15 min at 4 °C. The pellet was resuspended in buffer H and centrifuged at 800 × g at 15 min at 4 °C. The nuclear pellet was saved and the two supernatants were combined. The supernatant was then centrifuged at 10, 000 × g for 15 min at 4 °C. The pellet was resuspended in Buffer H and fractionated using a discontinuous sucrose gradient consisting of 0.85, 1.0, and 1.2 M sucrose at 100,000 × g for 2 h at 4 °C. After centrifugation, the synaptosomal and mitochondrial fractions were isolated at the 1.0/1.2 interface and the pellet respectively. The nuclear pellet was resuspended in Buffer H with 0.5% NP-40 and incubated on ice for 1hour. The sample was then centrifuged at 1,000 × g for 10 min. The pellets were washed with Buffer H with 0.5% NP-40 three times. Protein concentration was determined with a Pierce BCA protein assay (Life Technologies, Grand Island, NY).

### AHA protein enrichment for immunoblot analysis

The precipitated pellet was re-suspended in 8 M urea. This suspension was centrifuged, the supernatant was saved, and the resulting pellet was resuspended in 5% SDS and heated at 100C for 10 min. After heating, the suspension was centrifuged, and the supernatants were combined and enriched with Neutravidin beads for 2hours at room temperature while rotating. The beads were washed with PBS. The proteins were eluted with 4X Laemmli Sample Buffer (Bio-Rad) with β-mercaptoethanol, and the elution was used for immunoblot analysis.

### Immunoblot analysis

Tissue samples were solubilized with 4X Laemmli Sample Buffer (Bio-Rad) with β-mercaptoethanol , separated with 4–12% Bis–Tris gradient gel(Life Technologies), transferred to PVDF blotting paper, and developed as previously described^71^. The immunoblotting antibodies were β-actin(Sigma#A5441), CCT5(Scbt#sc-377261), CCT8(Scbt#sc-376188), COXIV(CST#4,850), EIF1A(CST#2,538), HDAC2(ProteinTech#12,922–3), and SynGAP(Abcam#ab3344). The immunoblots were quantitated as previously described^34^.

### Bioinformatic analysis

Protein function was assigned using Panther, as shown in Fig. S1B^72^. The slopes were calculated with the linear function: y = mx + b. AHA protein half-lives were estimated using a method similar to that presented by Dörrbaum et al.^73^. Specifically, average AHA protein ratios at each chase time point were calculated for the three mice. For each protein linear regressions were then performed on the ln-transformed average AHA protein ratios. If ${s}_{p}$ is the slope of the resulting linear function for AHA protein $p$, the degradation constant ${\lambda }_{p}$ is then estimated as ${-s}_{p}$. AHA protein half-lives (in days) ${T}_{{1/2}_{p}}$ are then estimated as follows:

$${T}_{{1/2}_{p}}=\frac{\mathrm{ln}(2)}{{\lambda }_{p}}$$

Transmembrane proteins (Fig. S3C) were determined using UniprotKB^74^. For the disorder correlation (Fig. S3D-F), Mobi-lite software was used to determine the Disorder Consensus^75^ and Esprite software was used to determine disorder from X-ray and NMR databases^76^. PST clustering analysis was performed using Ward’s algorithm^77^ and Euclidean distance as a distance measure between PSTs. This clustering analysis was implemented in R for dendrogram generation using the OompaBase v.3(https://oompa.r-forge.r-project.org/) and ClassDiscovery v.3 (https://www.rdocumentation.org/packages/ClassDiscovery) packages. Clusters were determined upon dendrogram visual inspection, the average slope, and shape trajectory. Localization analysis in Fig. 3A was performed by FunCoup v3.0^78,79^. Mitochondrial proteins in Fig. 3B,C were annotated using the UniprotKB database. Ingenuity Pathway Analysis(version: IPA Fall Release (September 2016); https://digitalinsights.qiagen.com/products-overview/discovery-insights-portfolio/analysis-and-visualization/qiagen-ipa/) was used to calculate significantly enriched cellular functions (Fig. S5A and B)^80^. For protein interaction analysis (Fig. S7A), the entire CORUM database was searched for complexes with at least two proteins from our data and redundant complexes were discarded. For the correlation matrix (Fig. S2), a Pearson’s correlation coefficient was calculated for all pair-wise comparisons between the 24 experiments using the log2 ratio values from the common proteins between each pair of experiments.

## Supplementary information

Supplementary file1

Supplementary file2

Supplementary file3
