# Supplementary material for: Quantitative analysis of global protein stability rates in tissues
Source: Sci Rep. 2020 Sep 29;10:15983. doi: 10.1038/s41598-020-72410-y (PMC7524747; doi:10.1038/s41598-020-72410-y)
Supplement: Supplementary file 1 — Supplementary file1 [file 41598_2020_72410_MOESM1_ESM.docx]

Quantitative Analysis of Global Protein Stability Rates in Tissues

Daniel B. McClatchy^1^, Salvador Martínez-Bartolomé^1^ , Yu Gao^2^, Mathieu Lavallée-Adam^1,3^, and John R. Yates III^1*^

^1^ Department of Molecular Medicine, The Scripps Research Institute, La Jolla, CA, USA

^2^ College of Pharmacy, University of Illinois at Chicago, Chicago, IL, USA

^3^ Department of Biochemistry, Microbiology and Immunology and Ottawa Institute of Systems Biology, University of Ottawa, Ottawa, ON, CAN

*Correspondence: [jyates@scripps.edu](mailto:jyates@scripps.edu)

**
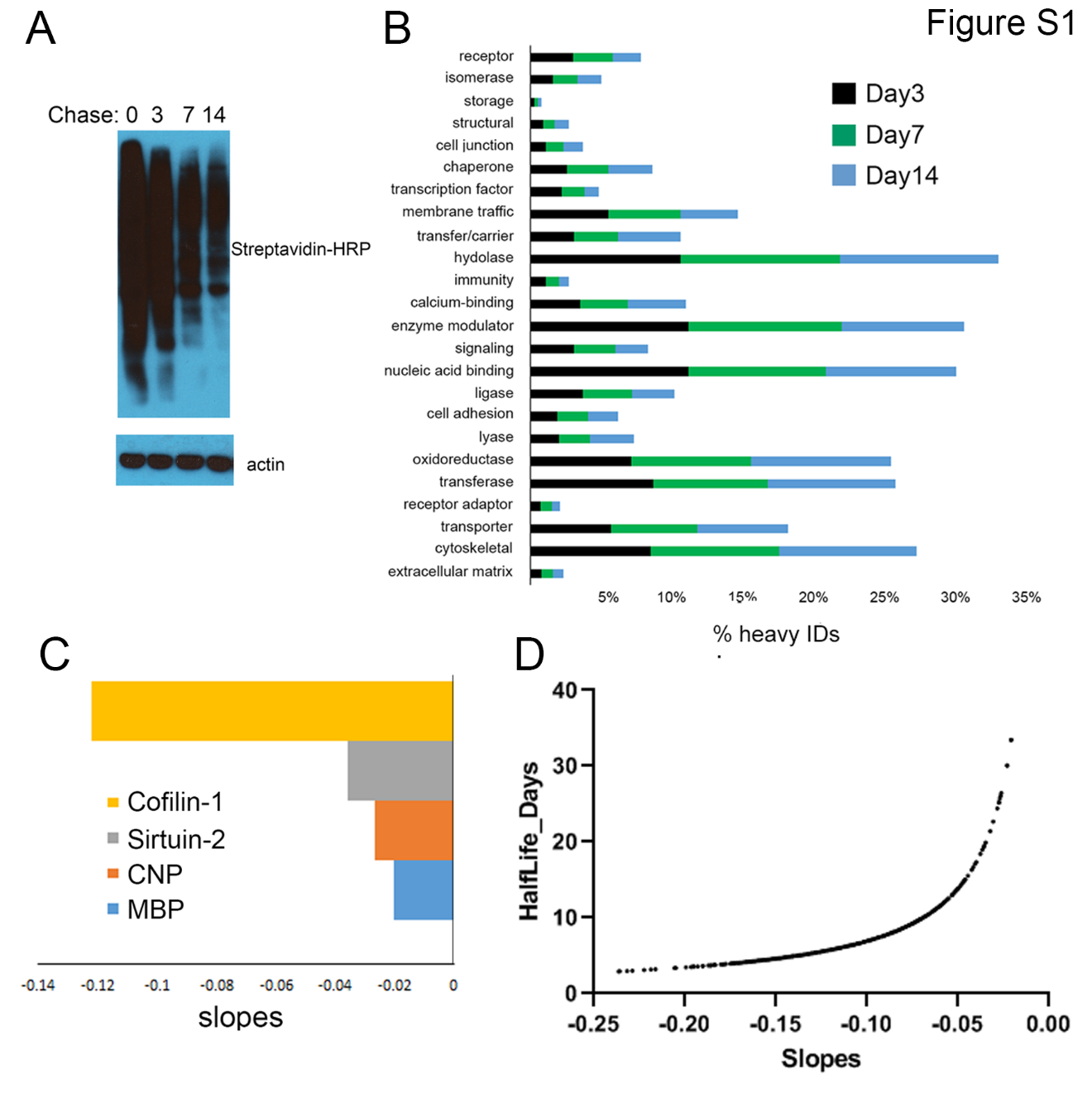
**

**Figure S1.** **A**, Immunoblot analysis of brain tissue demonstrates a decrease in detection of biotin-alkyne with increase chase time. The immunoblot was either probe with streptavidin-HRP or β-actin (loading control). The actin image is from a separate processed immunoblot. The uncropped actin immunoblot is in Fig. S9. **B**, Identical function protein classes were identified at different chase time points. Y-axis represents the percentage of genes from each dataset (i.e. Day3, Day7, Day14). **C**, Slopes of the PST correspond to previously published reports on protein stability using different methods. Y-axis is the slope. **D,** Correlation between slopes of PST and half-lives of the data from Fig.2A and 2B. Pearson’s correlation test determined a significant correlation (p < 0.0001) of r = 0.84. X-axis is the slope and y-axis is the half-life in days.

**
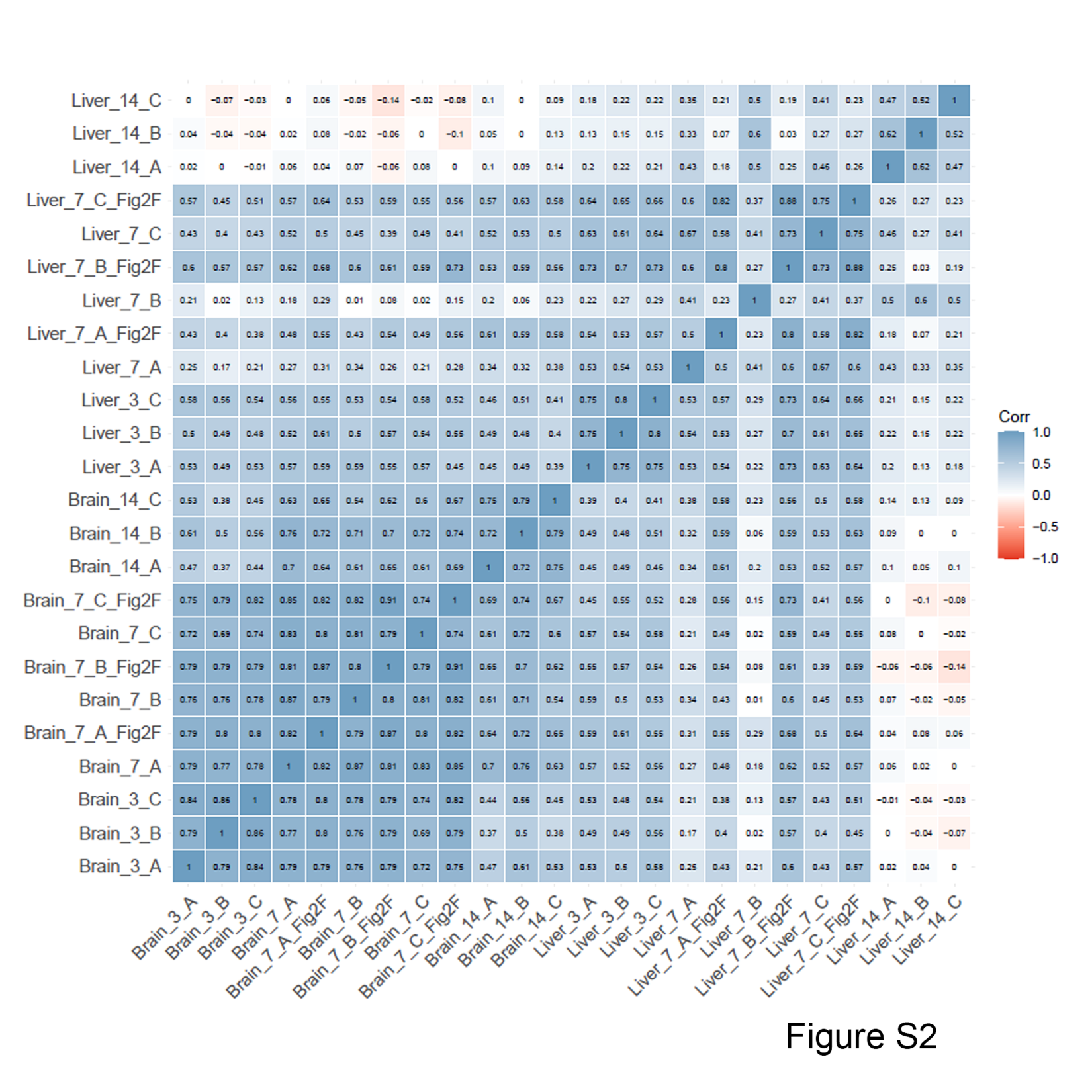
**

**Figure S2.** Correlation matrix between all brain and liver quantitative analyses at Day3, 7, and 14 shown in Fig. 2A and 2B. In addition, the brain and liver quantitative analyses at Day7 shown in Fig. 2F. Numbers in the matrix are Pearson’s correlation coefficient.

**
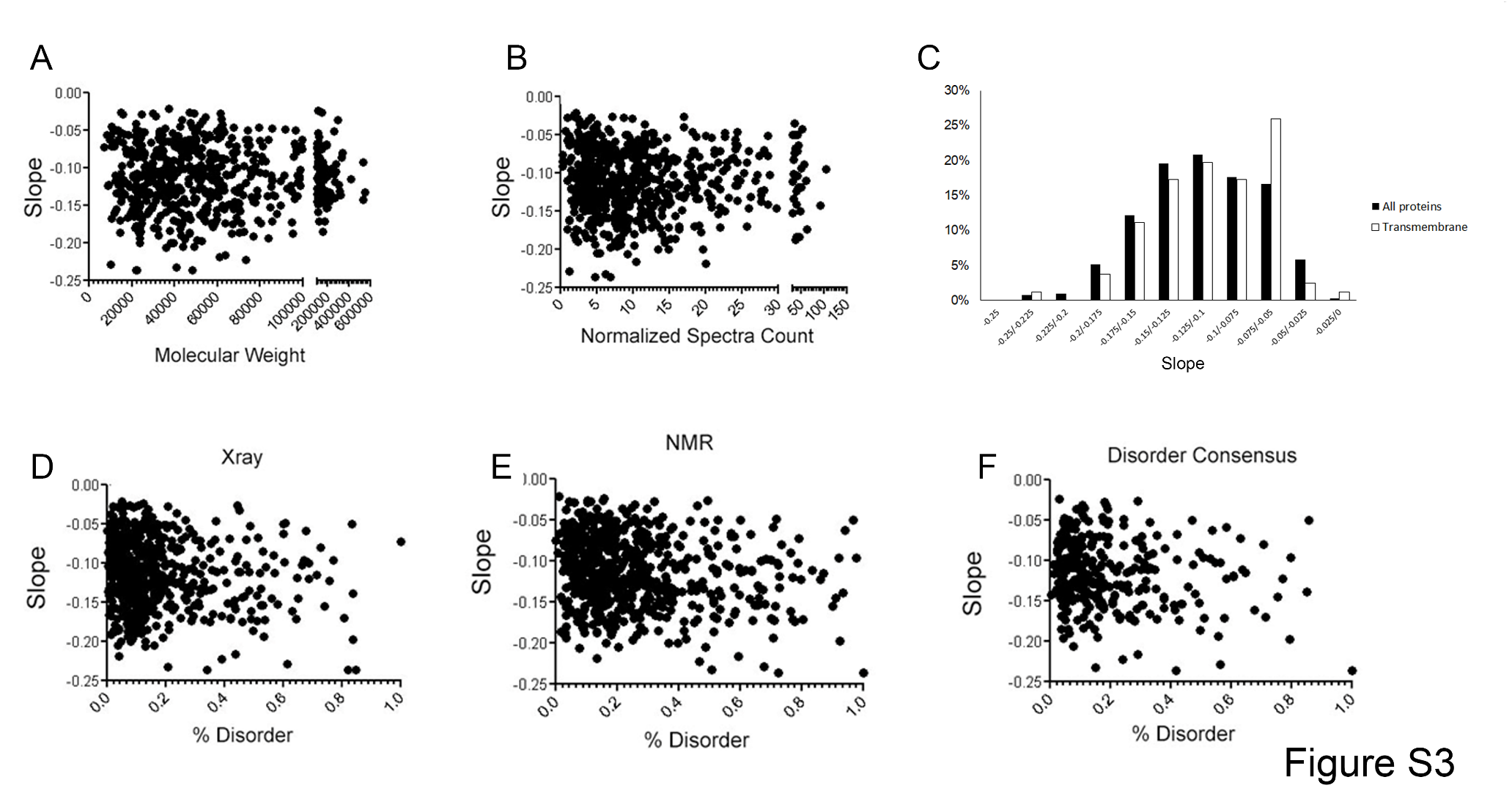
**

**Figure S3.** Correlation between protein stability and intrinsic protein characteristics using the brain dataset. There was no correlation with PST in the brain dataset with molecular weight (r = 0.12; p-value = 0.7639) (**A**), protein abundance (r = -0.003;p-value = 0.9481) (**B**), or membrane proteins(Kolmogorov-Smirnov test, p-value = 0.23)(**C**). There was a significant negative correlation with slope(y-axis) and percentage protein disorder predicted(x-axis) from Xray crystallography data (r=0.1551; p-value = 0.0001) (**D**), NMR data (r=-0.2091; p-value p-value < 0.0001) (**E**) or a multiple predictor disorder algorithm(r= -0.215; p-value = 0.0002 (**F**).

**
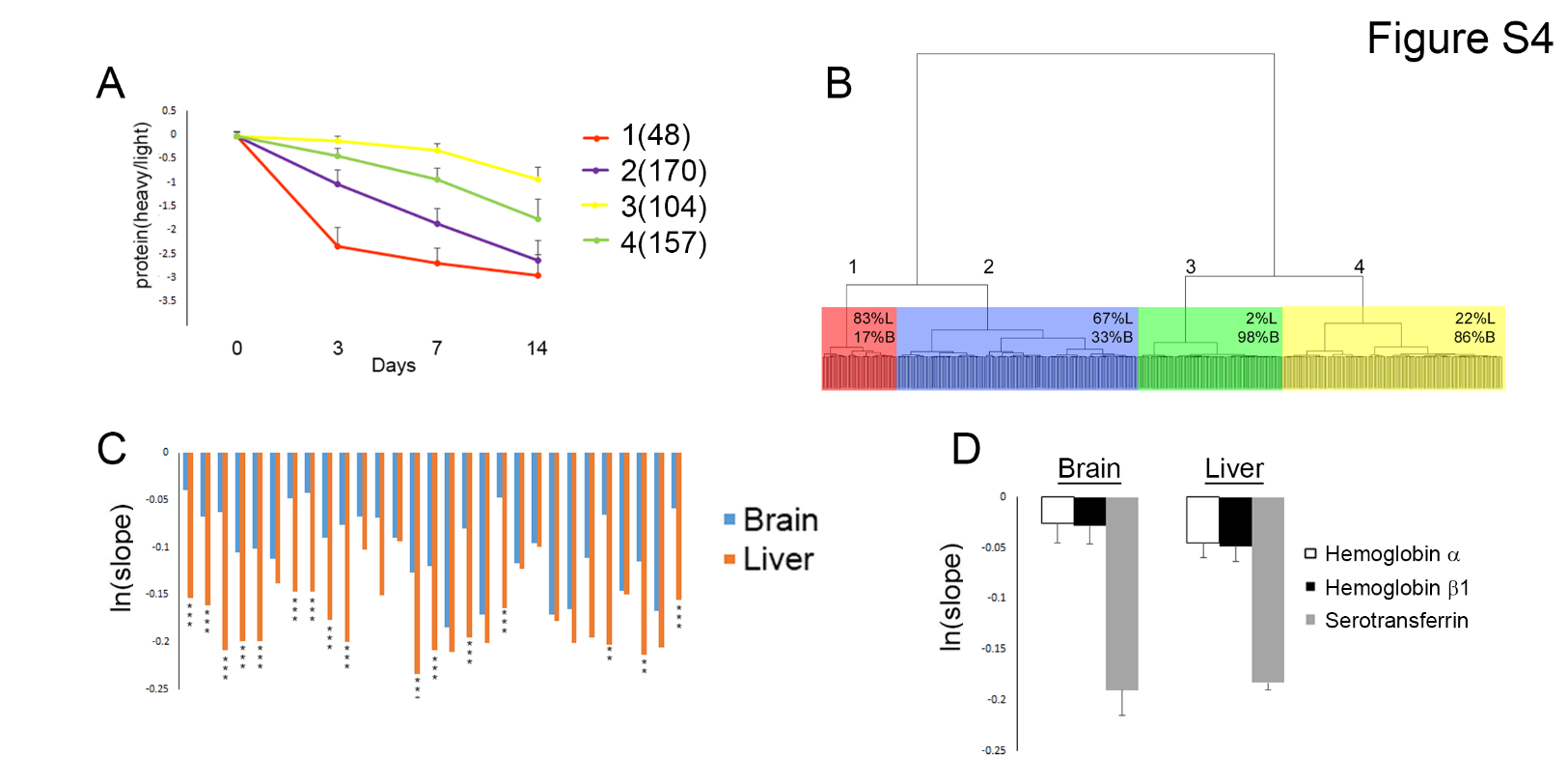
Figure S4. A**, Clustering analysis was performed on PST from liver and brain together. For each cluster, the average protein heavy/light ratio and standard deviation at each time point was plotted. The number of proteins in each cluster is in parentheses in the legend. **B**, Clusters in A are dominated by one tissue. The dendrogram of the PST summarized in A was annotated with the percentage of trajectories from liver(L) and brain(B) in each of the clusters. The dendrogram was generated using the OompaBase v.3(http://oompa.r-forge.r-project.org/) and ClassDiscovery v.3 (<https://www.rdocumentation.org/packages/ClassDiscovery>) packages. **C**, Statistical analysis of proteins quantified in both liver and brain but assigned to different clusters. Each blue(brain) and orange(liver) pair represent the same protein quantified in both tissues and the y-axis is the natural log transformation of the average slope. A two-tailed t-test was performed on each protein. **p < 0.01, ***p < 0.001. **D**, Blood and serum proteins had similar quantification results in both tissues. The natural log transformation of the average slope(y-axis) of the average of three biological replicates.

**
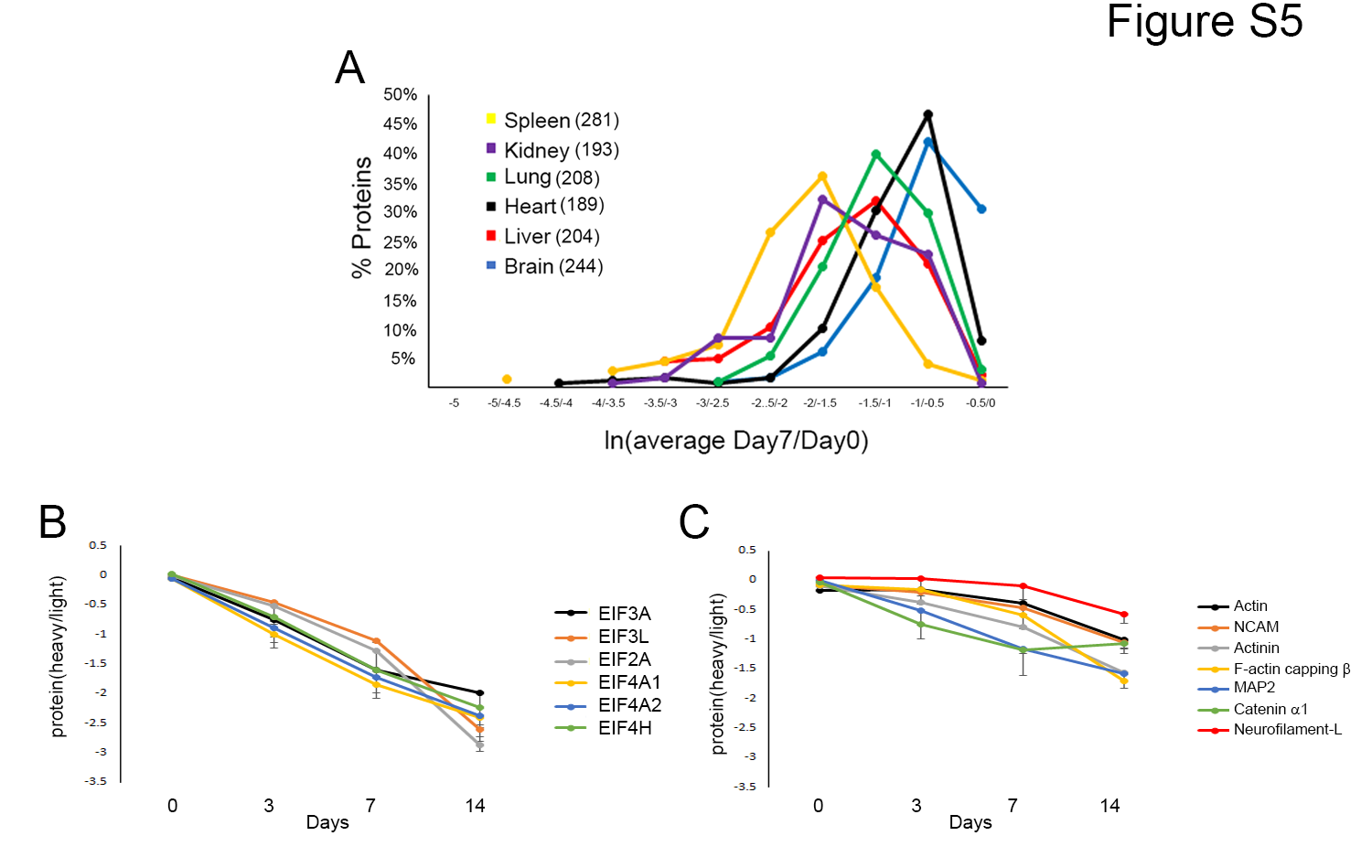
**

**Figure S5**. **A**, Distributions of Day7/Day0 protein averages for multiple tissues. Only proteins with coefficient of variation (CV) < 0.35 were plotted in the histogram. N=3 for all tissues except for spleen (N=2). The number in parentheses represents the proteins in each tissue distribution. Proteins involved in translation are enriched in the unstable protein dataset (**B**) and protein involved in the regulation of the cytoskeleton are enriched in the stable protein dataset (**C**). The average protein heavy/light ratio from biological replicates at each time point was plotted after a natural log transformation on the y-axis.


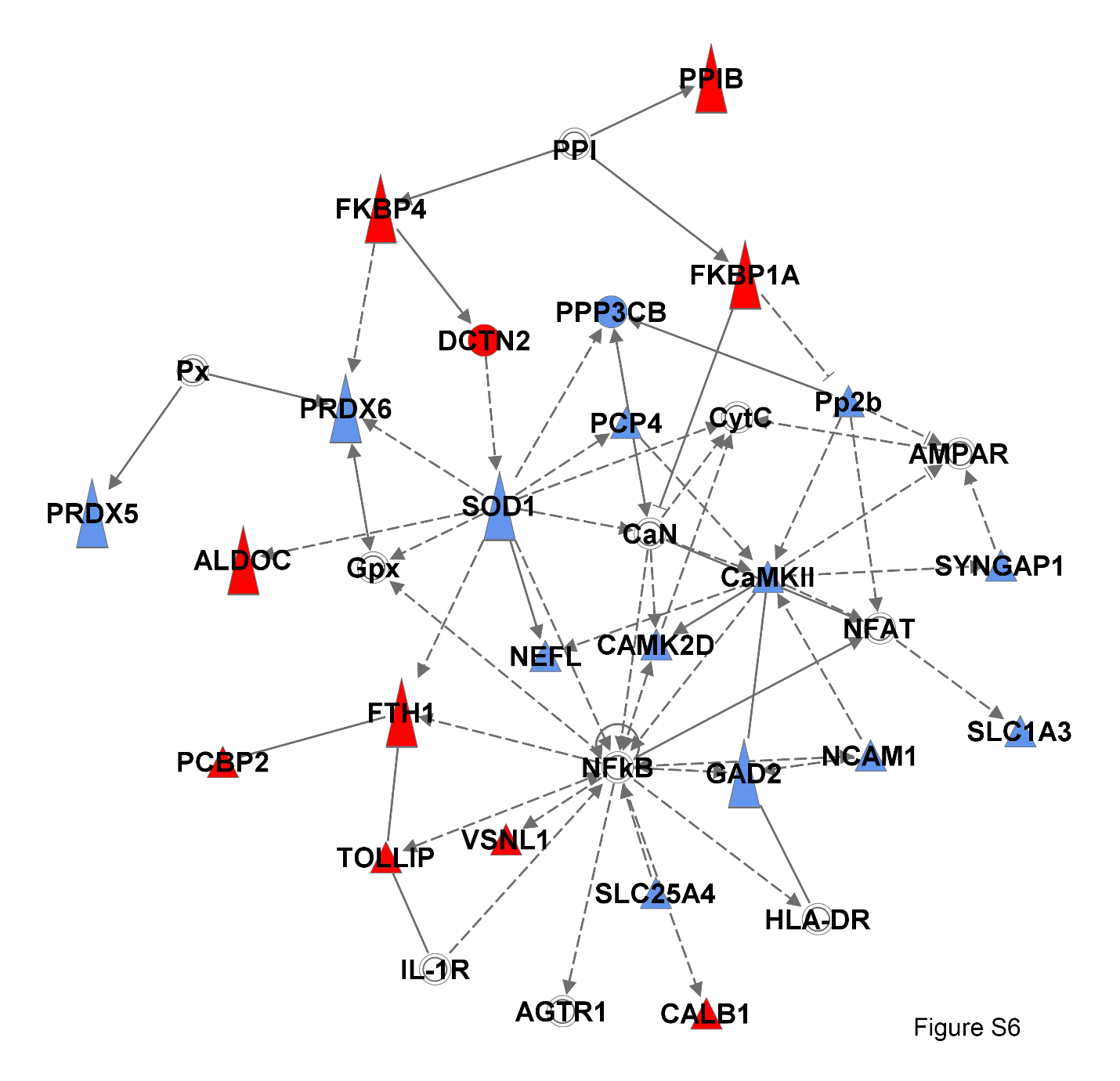


**Figure S6.** Stable(blue) and unstable(red) proteins form a signaling network that supports cell death. Image was created using Ingenuity. Solid lines represent direct interactions and dotted lines represent indirect interactions. White shapes represent proteins not quantified in the study. This figure was generated using Ingenuity Pathway Analysis(version: IPA Fall Release (September 2016); https://digitalinsights.qiagen.com/products-overview/discovery-insights-portfolio/analysis-and-visualization/qiagen-ipa/).

**
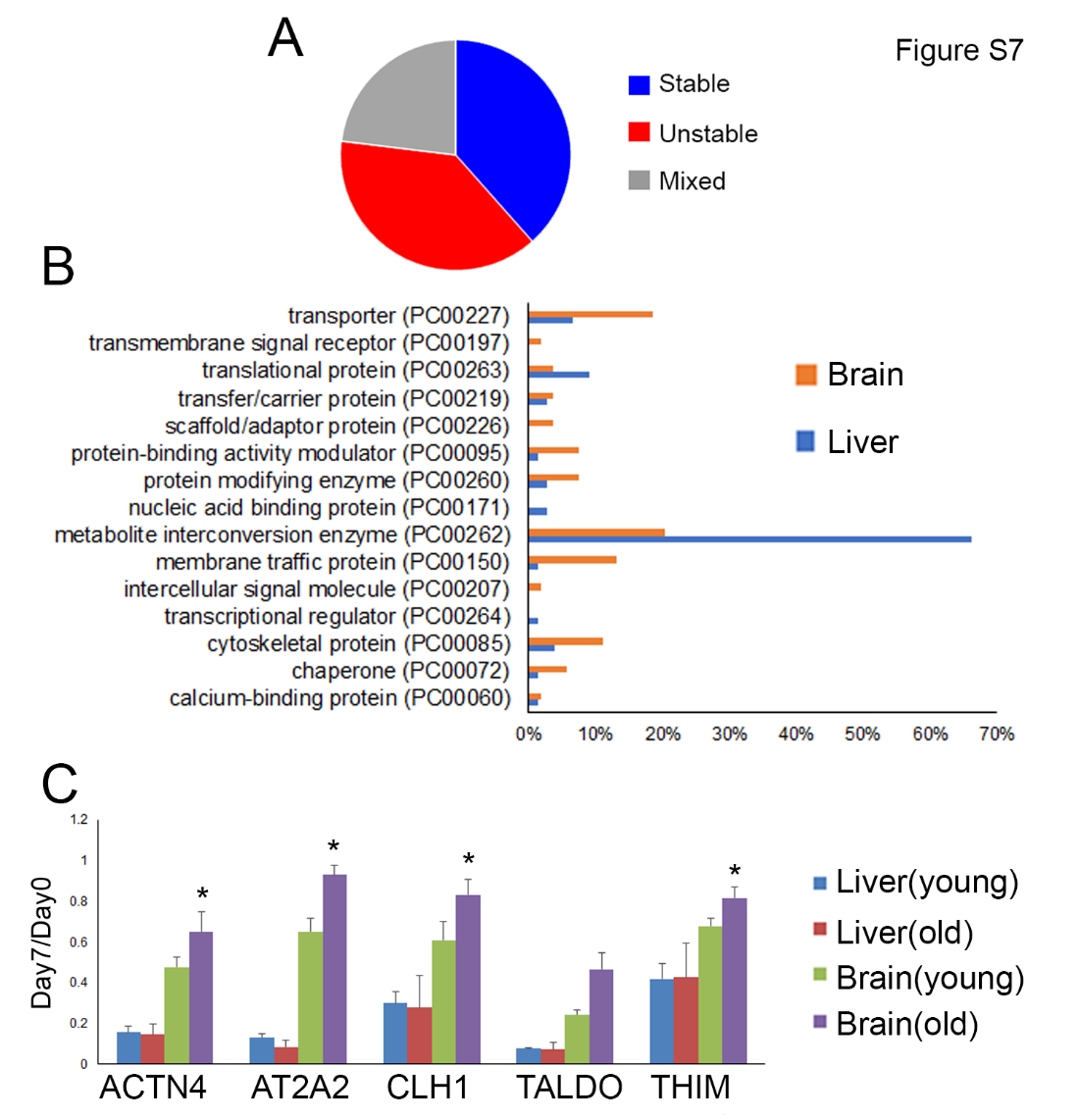
**

**Figure S7. A**, Protein complexes identified by the CORUM database using the proteins quantified in Fig. 2E. The percentage of complexes containing only stable proteins(blue), only unstable proteins(red), or a mixture of stable and unstable proteins(gray) is shown. **B,** Functional protein classes are shown in Fig. 4E and F. Protein function was determined by the Panther Classification System^81,82^. Only the proteins that were determined to be significant were used from the brain dataset while the entire liver dataset was used. X-axis is the percentage of proteins from each dataset annotated to each function class. The number in parentheses is the Panther class identification number. **C**, Proteins that were significant in the brain analysis in Fig. 4E and quantified in liver dataset in Fig. 4F. The gene names are on the x-axis and the y-axis is the average Day7/Day0 ratios.

**
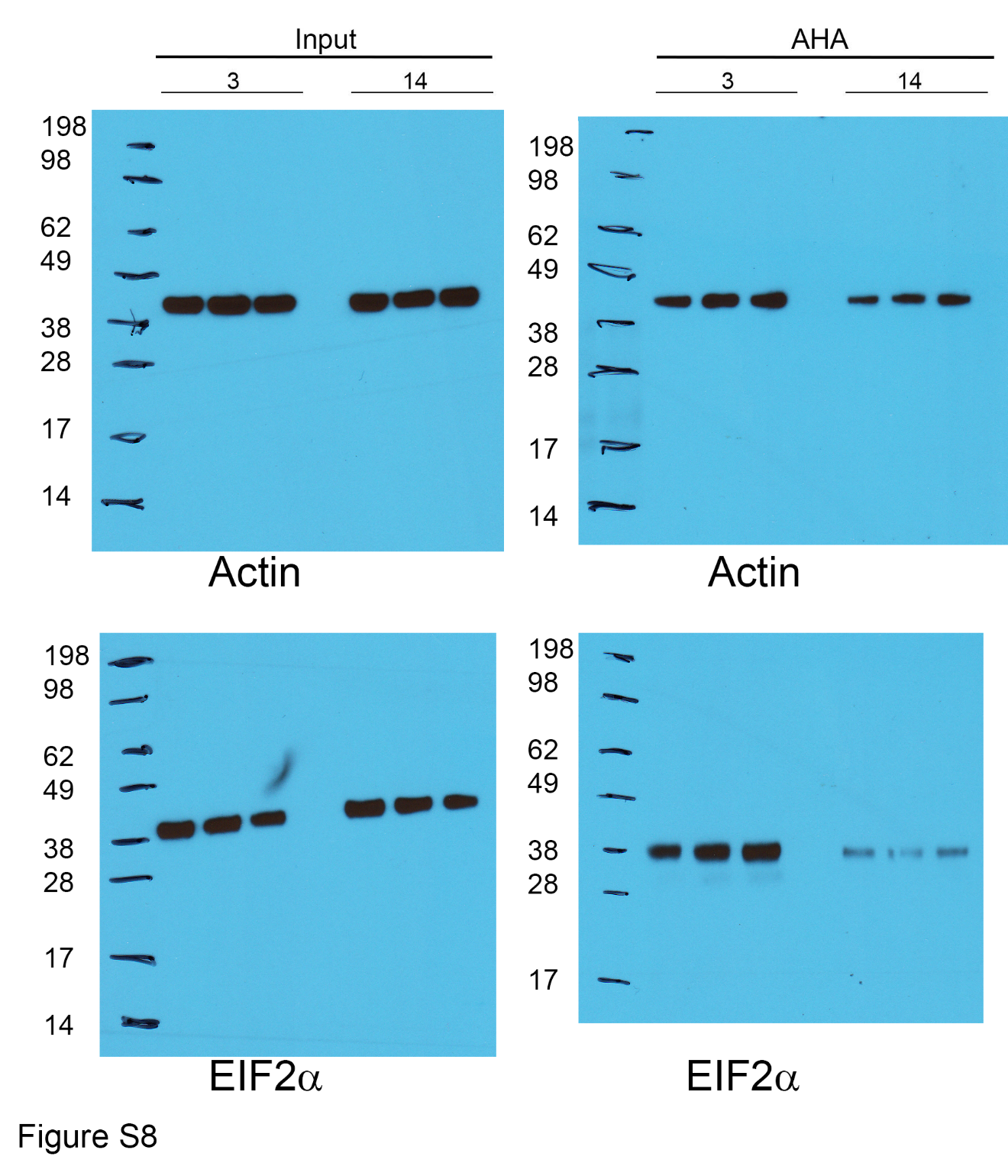
**

**Figure S8.** The uncropped immunoblots from Figure 3D.


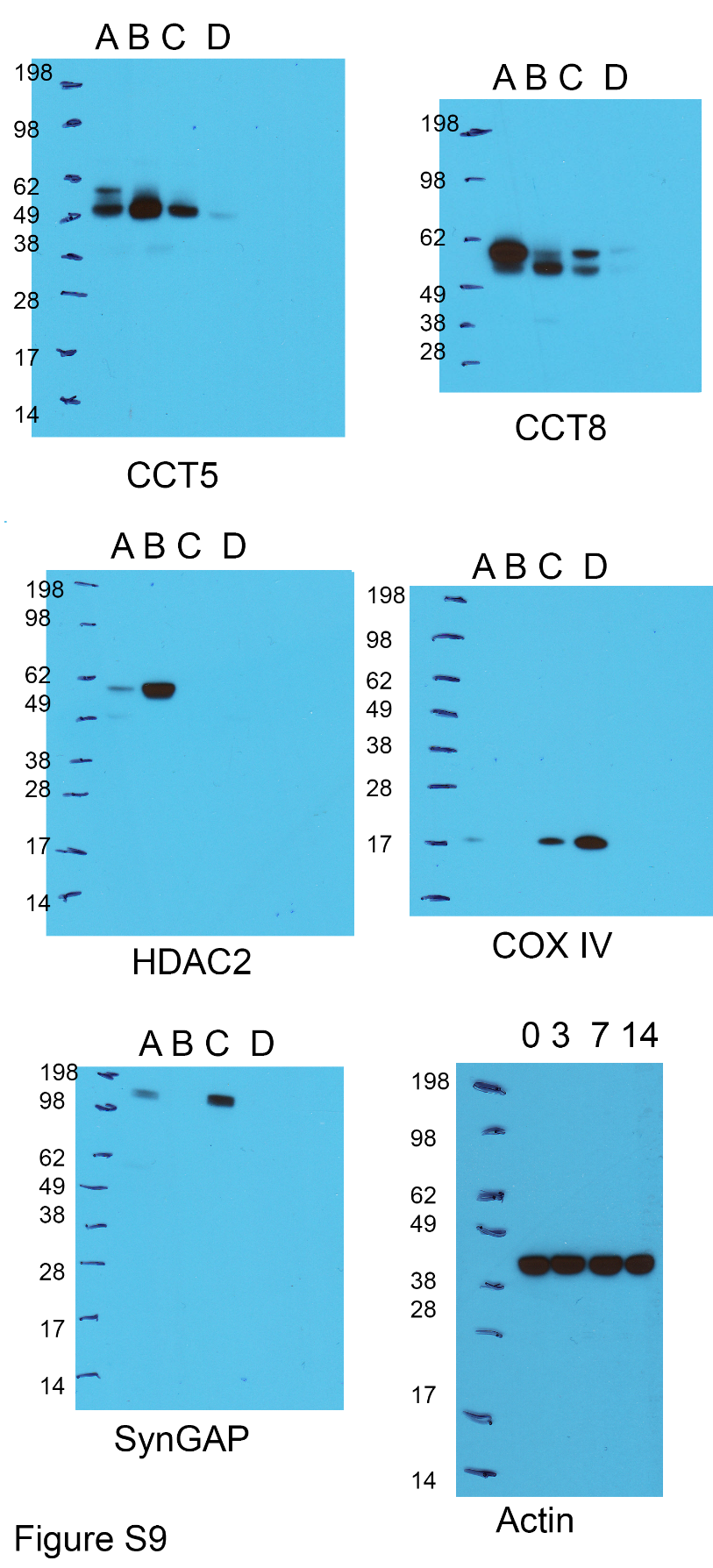


**Figure S9.** Uncropped immunoblots from Figure 4A(CCT5, CCT8,HDAC2, COX IV, and SynGAP) and from Figure S1A (Actin).
