# Supplementary material for: Quantitative analysis of global protein stability rates in tissues
Source: Sci Rep. 2020 Sep 29;10:15983. doi: 10.1038/s41598-020-72410-y (PMC7524747; doi:10.1038/s41598-020-72410-y)
Supplement: Supplementary file 2 — Supplementary file2 [file 41598_2020_72410_MOESM2_ESM.docx]

# Supplementary Data

*All raw MS data files are available on ProteomeXchange PXD015584.*

**TableS1**- All the proteins quantified from brain in all three mice at three timepoints(Day3, Day7, and Day14). Table contains the average heavy/light protein ratio in the mice, relative standard deviation(rsd) of each measurement, and slope of these averages for each protein. All data has been natural log transformed. Data plotted in Figure 2B.

**TableS2**- All the proteins quantified from liver in all three mice at three timepoints(Day3, Day7, and Day14). Table contains the average heavy/light protein ratio in the mice, relative standard deviation(rsd) of each measurement, and slope of these averages for each protein. All data has been natural log transformed. Data plotted in Figure 2A.

**TableS3** – Proteins with their calculated slopes and half-lives. Data is depicted in Fig. S1D.

**TableS4** - Proteins from brain with their assigned cluster in Figure 2E

**TableS5** – Proteins from liver with their assigned cluster in Figure 2D

**TableS6** - Proteins from liver and brain with their assigned cluster in Figures S4A and S4B.

**TableS7** – The proteins quantified in both liver and brain and the statistical information for Figure S4C.

**TableS8-** Proteins quantified from different tissues from 3 different mice for Figure 2F and S5A.

**TableS9**– Statistical results from Tukey’s multiple comparison test of the data in Fig. 3C.

**TableS10**– Significantly enriched cellular functions in the unstable brain dataset in Figure S5B.

**TableS11**- Significantly enriched cellular functions in the stable brain dataset in Figure S5C.

**TableS12** – Protein complexes identified in the CORUM database. Data found in Fig. S7A.

**TableS13**- The proteins quantified in Figure 4E. The average Day7/Day0 ratios quantified in three 2-month and three 1-year mouse brains.

**TableS14**- The proteins quantified in Figure 4F. The average Day7/Day0 ratios quantified in three 2-month and three 1-year mouse livers.
